# A theoretical framework for early human studies: uncertainty, intervention ensembles, and boundaries

**DOI:** 10.1186/1745-6215-13-173

**Published:** 2012-09-22

**Authors:** Jonathan Kimmelman

**Affiliations:** 1 Biomedical Ethics Unit/Experimental Medicine, McGill University, 3647 Peel Street, Montreal, QB H3A 1X1, Canada

**Keywords:** Phase 1, Phase 2, Research ethics, Risk, Uncertainty, Dose escalation, Drug development

## Abstract

Clinical development of novel therapeutics begins with a coordinated sequence of early phase clinical trials. Such early human studies confront a series of methodological and ethical challenges. In what follows, I propose a theoretical framework for early human studies aimed at informing the negotiation of these challenges. At the outset of clinical development, researchers confront a virtually undifferentiated landscape of uncertainty with respect to three variables: outcomes, their probability of occurrence, and operation dimensions needed to effectuate favorable outcomes. Early human trials transform this uncertain landscape into one where there are grounds for belief about risk and benefit for various combined operation dimensions. To accomplish this, studies set out with two aims. First, they identify a set of operation dimensions that, when combined as a package (intervention ensemble), elicits a reasonable probability of a target outcome. Second, they define the boundaries of dimension values within an intervention ensemble. This latter aim entails exposing at least some volunteers in early studies to treatments that are inactive or excessive. I provide examples that illustrate the way early human studies discover and delimit intervention ensembles, and close by offering some implications of this framework for ethics, methodology, and efficiency in clinical development of new interventions.

## Review

### Introduction

Initial tests of new therapeutic strategies in human trials are a key step in clinical translation. While widely viewed as necessary for drug development, they frequently draw debate about design, risk, and subject protection. Do phase 1 studies that enroll patients with advanced disease ‘collude’ with unrealistic expectations of desperate volunteers [1]? Should protocols enrolling patients employ designs that maximize the chances for patient benefit [2]? Should phase 1 studies be presented to cancer patients as a treatment option [3]?

Addressing these questions and evaluating specific protocols for ethical and scientific merit should start with an accurate description of what early human studies (commonly glossed as phase 1 and 2 trials) are, and what they set out to accomplish. In what follows, I build on prior theoretical accounts of early human studies and propose a framework for understanding their goals and methods. The ethical planning and implementation of early studies requires coordinating a series of investigations of an intervention with each other; the analysis offered below aims to provide greater purchase on various ethical, technical, and coordination problems encountered in early human studies.

### Premises and terminology

Developing a theoretical framework of early human trials requires clarifying terminology. In what follows, I use ‘early human trials’ to capture a large family of study types, including what are variously called phase 0 trials, pilot studies, translational trials, feasibility studies, phase 1 trials, dose-ranging trials, dose-finding trials, and many kinds of phase 2 trials.

I distinguish ‘early human studies’ from ‘phase 1 trials’. The latter is a regulatory term, rather than a methodological or scientific one. It has a strict definition directed towards regulatory objectives [4]. It thus excludes many human investigations that share similar overall objectives, but are unregulated (e.g. initial tests of surgeries) or that go under different labels.

Early studies involve a sequence of investigations rather than a single test. Multiple phase 1 trials, aimed at answering different questions, often precede phase 2 trials, and new phase 1 trials are often conducted long after a drug has completed phase 2 trials. For example, the cancer drug erlotinib was first introduced into phase 1 trials before 2001 [5], yet since approval in 2004, phase 1 trials targeting new indications [6] or in combination with other approved anti-cancer agents have been conducted [7]. ‘First in human’ trials, the most stereotyped category of phase 1 trials in the ethics literature, represent a minority of all early human trials.

Many early human trials are described as having ‘dose’ as the independent variable and ‘safety’ as the dependent variable. However, early human trials manipulate variables other than dose, especially in the context of complex interventions. And they observe other sorts of dependent variables. For example, early studies collect provisional indication of biological activity, often through pharmacodynamic outcomes; investigators need to know something about response in order to design later trials.

In sum, early human trials involve a suite of investigations, all of which gather evidence about different aspects of the intervention. I begin this analysis with the uncontroversial ethical premise that a suite of early studies ought to minimize the number of subjects, and their level of risk, for gathering adequate evidence to support late-phase, confirmatory trials.

### Early human trials and uncertainty

Early human trials are conducted at the point of highest uncertainty in clinical development of an intervention. As noted elsewhere, these uncertainties are different in character from those encountered in late stages of intervention development [8]. Uncertainties in late stages can be articulated in terms of a defined set of variables, discrete outcomes, and narrowly bounded estimates of probabilities. For instance, investigators anticipate certain kinds of toxicities and clinical responses, and they can assign probabilities for each. In early stages of intervention development, investigators encounter uncertainty. Decision science often calls the former kind of uncertainty ‘risk’, and the second, ‘ignorance’. As Djulbegovic observes, uncertainties encountered in drug development have a strict temporal relationship with each other: ignorance about new drugs must be resolved before researchers can address more discrete questions of risk. Research designs should be suited to the type of uncertainty encountered by drug developers [8,9]. Accordingly, the goal of early human studies is to transform ignorance in early stages of drug development into risk by identifying conditions where there are reasonable grounds for believing a drug has clinical utility [8]. The goal of later phase trials is to reduce this risk by rigorously confirming clinical utility using randomized trial designs.

Early human studies confront three forms of ignorance. The first is outcome uncertainty. When initiating human studies, investigators often have little basis for anticipating many toxicities. They may even have a foggy view of what aspects of disease will respond. The second uncertainty is probabilistic. In early human studies, investigators are unable to assign reasonably bounded probabilities for those outcomes that are anticipated.

The third uncertainty concerns intervention activities, which I hereafter call operation dimensions. New drugs, biologics, surgeries, et cetera only acquire therapeutic activity by coordinating them with various other practices, knowledge, and materials (an ‘intervention ensemble’). For example, at the point human investigations are initiated, researchers do not yet know which dose of drug to apply, or how frequently to apply the treatment. Some dimensions, like dose, are known in advance to be consequential even if their values are not known. Other dimensions are only discovered in the course of human studies. The discovery of unexpected cardiotoxicity in early human studies, for example, can lead researchers to discover concurrent cardiac monitoring as a necessary operation dimension for safe and effective intervention.

The uncertainties confronted in initiating human studies are not boundless. Preclinical studies, prior clinical evidence, and knowledge of biology often provide grounds for believing that, say, picomolar quantities of drug will not cause toxicity. Within these wide bounds, however, investigators confront a virtually undifferentiated landscape of uncertainty with respect to a dimension’s value given a target outcome. Thus, for example, before early human investigations are conducted, investigators have no solid grounds for believing that any one dose will outperform another with respect to risk/benefit. Early human investigations set out to transform this landscape of uncertain outcomes, probabilities, dimensions and values into one where there are grounds for belief about the risk/benefit balance for an intervention ensemble.

This transformation entails two complementary research practices. First, investigators set out to identify a set of operation dimensions (that is, the identity of the dimensions and values) that, when combined as a package, elicits a reasonable probability of a target outcome, such as disease response with acceptable toxicity or serum levels of a drug with acceptable toxicity. For example, early human studies aim at determining dose, schedule, and the patient group in whom an anti-cancer drug is likely to produce tumor shrinkage with acceptable toxicity. This process of clarifying operation dimensions within an intervention ensemble is necessary for advancing a drug to later stages of clinical testing, because it would be costly and burdensome to use large populations in late human trials to test intervention ensembles that do not have a defined and favorable probability of achieving a target outcome.

Second, investigators set out to define the boundaries of dimension values in this intervention ensemble. That is, early human investigations clarify dimension values that demonstrably belong outside a space of combined operation dimensions that produce an intended risk/benefit. This latter process is just as crucial as the first: it allows investigators to better plan subsequent trials, and it resolves important questions for clinical practice. This process of defining boundaries is ethically nettlesome, because it necessarily entails exposing some patients to ineffective and/or unsafe intervention ensembles.

Consider a drug given to patients to prevent neuronal injury following stroke. One key dimension for eliciting clinical utility is timing of administration. If investigators fail during clinical development to ever clarify the latest time after stroke in which the drug provides neuroprotection, investigators risk either designing late phase trials in which real treatment effects are diluted due to implementation of a wide window of drug delivery, or they risk designing needlessly rigid and expensive phase 2 studies that replicate precisely dimension values identified in phase 1. In any event, if the drug is shown effective in late phase trials, failure to define boundaries leaves physicians and healthcare systems uncertain about how best to apply an intervention.

Early human studies do not resolve all uncertainties. They do not define boundaries for every relevant dimension value in an intervention ensemble. There can be practical reasons why boundary values of certain dimensions are important to learn early in development. There can also be practical reasons where knowing boundaries is less important or impossible to clarify. This might occur if a drug is very safe and cheap, or if the fall-off of risk-benefit outside the therapeutic window is believed to be so steep that delivery of drug outside the window to establish boundaries would be unethical. Some boundaries are clarified only in later stages of development, and the precise boundaries of an intervention ensemble can be impossible to resolve in early human studies, because the signal needed to resolve some uncertainties can require large sample sizes. Early studies often lack the statistical power to optimize dimension values inside the intervention ensemble. Instead, the goal of early human studies is to find at least one intervention ensemble that is a compelling candidate for confirmation in late phase trials.

This account of early phase studies builds on various themes in the methodological literature on phase 1 trials. Steven Piantadosi [10] and Benjamin Djulbegovic [8], for example, describe translational trials and early phase trials, respectively, as aimed at reducing uncertainty. The former describes ‘translational trials’ as aimed at information gain concerning drug properties, defined formally in terms of entropy, rather than demonstration of clinical promise: ‘Many clinical outcomes [in translational trials] are equally informative about the treatment effect, though not equally promising’ (p215). Djulbegovic describes phase 1 and 2 trials in terms of helping to shape uncertainty about a new drug.

**Figure 1:**
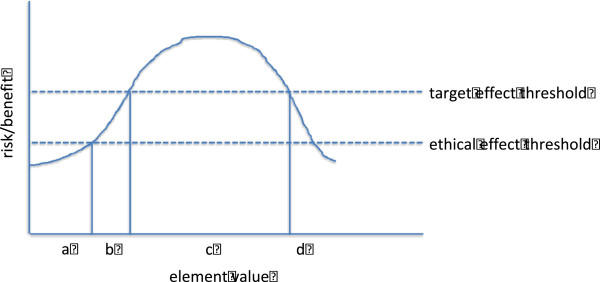
**How early trials proceed with respect to a single dimension. **Investigtors do not yet know the value for a given dimension, such as dose. (**A**) The investigators begin the study at a value that exceeds an ethical threshold (that is, value for which there is warranted belief that knowledge gain will redeem burdens and risks of drug administration). (**B**) Investigators escalate dimension values and cross a target effect threshold (which could be a pharmacokinetic variable, or a biological response of some sort). They have now defined the lower edges of dimension values. (**C**) They continue escalating and eventually re-cross a target effect threshold (**D**). When there are solid grounds for knowing they have crossed this target effect threshold, but well before re-crossing the ethical threshold, they discontinue escalation. The burdens or risks in (**D**) are considerably higher than elsewhere in the study, but enable warranted belief about the upper boundaries of therapeutic dimension values.

Though various commentators [11] discuss the necessity of identifying ‘optima’, ‘inflexion points’, or ‘bracketing the anticipated optimal dose’ [10], boundary identification across different dimensions, and assembly of intervention ensembles, is not a major focus of prior accounts of early human studies. The description offered here, depicted graphically for a single dimension in Figure 1, builds on prior accounts by drawing attention to the problem of maximizing the efficiency with which operation dimensions are identified, and boundaries defined across a series of early human studies. Maximizing efficiency of intervention ensemble definition is desirable because it minimizes the number of subjects exposed to inactive or ineffective interventions in drug development. For example, if early human trials in stroke fail to define the appropriate time for delivery of a neuroprotective drug, many patients in later human trials will likely be exposed to inactive application of the drug. Maximizing is also critical for minimizing expenditure of scarce resources, like equipment or investigator expertise, during clinical translation.

### Early human trials in practice: examples

Dose provides the clearest illustration of how early human trials define dimension and their value boundaries. Typically, a phase 1 trial begins by delivering drug at the lowest plausible level where a biological effect, such as a toxicity, might be observed. Studies administer escalating doses until toxicities are observed. Target modulation or a surrogate of efficacy might be concurrently monitored. Volunteers receiving insufficient or toxic doses define the boundaries of the dose dimension for further clinical testing. Nevertheless, dose is but one dimension that is explored through early human trials (see Table 1 for examples).

**Table 1:** Common dimensions in intervention ensembles

	**Examples**	**Cetuximab**	**Belimumab**	**Gene transfer for AID-SCID**
*Treatment dimension*				
Dose	mg/kg; cells; vector particles…	250 mg/m^2^	10 mg/kg	≥8 × 10^6 cells
Schedule	Daily; 4× weekly w/2 wk holidays…	Weekly until disease progression	3× @ 2-wk intervals; 4-wk intervals after	
Administration/target	Infusion; oral; intratumoral…	Intravenous infusion	Intravenous infusion	Intravenous infusion
Co-intervention	Radiotherapy; immunosuppresion…		Standard of care	Non-myeloablative conditioning
Risk mitigation	Cardiac monitoring; liver enzymes…	Serum electrolytes monitoring	Monitor patients for hypersensitivity	
Timing delivery	Symptom onset; 12 h after event…	After irinotecan or oxaliplatin failure		3 weeks after stopping PEG-ADA
*Population dimensions*				
Diagnostic criteria	Marker positivity; diagnostic score…	KRAS mutation in codon 12 or 13	α-nuclear Ab titer ≥ 1:80 and/or α-dsDNA	
Contraindications	Concurrent infection; clothing disorder…		Prior anaphylaxis to belimumab	Neutralizing Abs to vector (AAV8)
Indications	Disease; injury; at-risk population…	Metastatic colorectal cancer	Active systemic lupus erythematosus (SLE)	ADA-SCID
Age		Adults	Non-geriatric adults	Children
*Outcome dimensions*				
Endpoint	Change on scale; time to event…	Survival	SLE responder index	Immune system reconstitution
Duration	Short term; ‘long term’ remission…		1 year	Permanent

#### Therapeutic co-interventions

Many drugs require co-interventions to unlock their clinical utility. Early human trials provide an opportunity to identify these co-interventions and their dimension values.

Telaprevir was developed as an inhibitor of hepatitis C virus replication. Initial phase 1 studies in patients with chronic disease found that, when given at a dose of 750 mg, telaprivir reduced viral load. However, viral load rapidly rebounded in most patients. A later small, single-arm, early human trial tested telaprevir in combination with two standard treatments for chronic hepatitis C: pegylated interferon alpha 2a and ribavirin [12]. Patients receiving interferon and ribavirin treatment showed a sustained viral response after receiving telaprevir. Now, the question was how long to give each co-intervention, and whether one could be dropped. Variations of the combination therapy were tested in phase 2 studies. These confirmed the utility of combining all three drugs. The combination therapy later demonstrated efficacy in phase 3 trials [13].

#### Populations

Often, anticancer drugs enter development without a clear picture of which malignancies will respond. To discover the malignancy types to target in later trials and the ones to exclude, early phase trials sometimes sample patients with various malignancies.

Sometimes, however, early human trials are not executed in a manner that establishes boundaries for a target population. Consider the case of cetuximab, a monoclonal antibody that binds to and inactivates the epidermal growth factor receptor (EGFR) [14]. Molecular knowledge led investigators to pursue all prelicensure trials in colorectal cancer patients with EGFR-expressing tumors. The agent received regulatory licensure in 2004. Retrospective analysis of phase 2 results, however, did not show consistent relationships between EGFR expression and clinical activity. Some medical centers thus began offering cetuximab off-label to colorectal cancer patients with non EGFR-expressing tumors. Retrospective review of patient outcomes confirmed that, contrary to the FDA label, cetuximab response bore no relationship with EGFR expression [15]. In this episode, there were theoretical reasons to limit trials to patients expressing EGFR. However, by not testing this theory in prelicensure trials, drug developers missed opportunities to define boundaries of the target population. Though cetuximab was licensed successfully, this failure to define boundaries exacted inefficiencies for healthcare systems and practitioners, who confronted uncertainties surrounding the right patient population for this very expensive but potentially life-saving drug.

#### Delivery

Treatments often raise questions about how best to deliver an intervention. Throughout the 1990s, several teams claimed to have stabilized Parkinson’s disease by striatal implantation of fetal tissues. The first two randomized, sham controlled trials of the approach, however, failed to demonstrate statistical significance on the primary endpoint. They also uncovered previously unrecognized graft-induced dyskinesias [16]. A decade later, investigators are proposing to reinitiate transplant trials [17].

Among the many outstanding dimensions of the strategy is delivery. Unsuccessful randomized studies implanted cells in the striatum. However, some early studies support the targeting of structures adjacent to the striatum. Another unresolved issue is whether the entire striatum should be targeted, or merely structures within the striatum. Still another outstanding uncertainty concerns the type of cannula used to deliver cells: too large a diameter damages the site of delivery, which leads to death of transplanted cells; too small a cannula necessitates single-cell suspensions of fetal tissue, which can also lead to cell death [18]. Given the high level of neurosurgical risk and the scarcity of fetal tissues, an important task for early human studies will be to clarify the gross advantages for using particular techniques of delivery. This uncertainty might be resolved within a single protocol, or by running several parallel studies using different techniques [17]. Of course, coordinated studies or single studies aimed at resolving values for multiple dimensions entail non-trivial costs in terms of logistics and statistical efficiency.

#### Endpoints

Early human trials also clarify the kinds of endpoints sought in later trials. Belimumab is the first drug ever approved specifically for the treatment of systemic lupus erythematosus. The first phase 2 trial of belimumab failed to meet the co-primary endpoints of disease activity at 24 weeks and time to first flare during the 52 weeks. However, re-analysis of trial data indicated that a subset of patients, those with B-cell dysfunction, had indeed improved using a different measure of lupus response, the SRI. Human Genome Sciences then designed two phase 3 studies enrolling only patients with B-cell dysfunction, and using SRI as the primary endpoint. Both studies met their primary endpoints, leading to FDA approval [19].

Many newer, targeted, cancer drugs work by inhibiting tumor growth [20]. Standard criteria for measuring anti-cancer activity, like RECIST, can fail to detect otherwise potent activity. There is no reason why phase 1 trials in cancer or other disease areas cannot be used to explore a series of different endpoints so that the most appropriate ones are selected for primary endpoints in phase 2 trials [21]. Pharmacokinetic and pharmacodynamic endpoints can also provide crucial inputs for selecting values on dimensions like dose, schedule, or delivery.

## Discussion

To examine how this framework might assist decision-makers with negotiating ethical and methodological problems in testing new interventions, consider three challenges: ethical justification of risk, subject selection, and study design.

Some commentators contend that risks in phase 1 trials can be justified by therapeutic value. This carries several practical implications, for example, that trials can be presented to volunteers during informed consent as therapeutic opportunities. The analysis provided above reinforces criticisms of this position. As noted above, a key aim of early phase studies is establishing boundaries on values of each dimension. Occasionally, there can very compelling and reliable grounds for knowing these boundaries *a priori*. More typically this is not the case, and the task of boundary definition imposes a requirement that some patients receive inactive and/or harmful intervention ensembles. During the course of drug development, some studies should expose patients to inactive or excessive doses, and some patient classes should be uncovered as ineligible for future investigations of the intervention ensemble. Though investigators should not know in advance which patients will receive harmful intervention ensembles, they should expect that if a study has been properly implemented, at least some patients will be exposed. As argued elsewhere, [22] because the principle of justice dictates that harm to one patient cannot be ‘purchased’ with benefit received by another, risk in early human studies cannot be justified by appeal to therapeutic benefit. This view is captured by John O’Quigley’s observation that ‘in the areas of phase 2, 3 and 4 studies, the scientific approach guiding the statistician… and guiding the clinician are in… harmony. This is not so… for phase 1 dose-finding studies’ [11]. I would suggest this argument also holds for phase 2 studies that are still searching for dimension values, as when studies randomize patients to two different doses.

A second ethical and design problem in early human trials is subject selection. Geron’s phase 1 trial testing GRNOPC1, an embryo-derived treatment candidate for spinal cord injury, was controversial. Some criticized Geron for enrolling patients with recent injury, since a small probability of modest spontaneous recoveries might be abrogated by side effects of cell delivery [23]. Others countered that the subject selection was sensible, since patients with recent injury were more likely to benefit [24]. The analysis provided above can help address this dispute. At the point of trial initiation, Geron investigators confronted numerous second order uncertainties: how should cells be delivered? Can transient immunosuppression prevent rejection of delivered cells? What dose of cells can be delivered safely? Can cells stimulate recovery? The key question for trialists was how such uncertainties could be resolved with the lowest risk and smallest number of patients exposed to identify an intervention ensemble that could be advanced to randomized trials. If key uncertainties like safety, delivery, and immunosuppression could be reasonably resolved in patients at lower risk, Geron might have minimized risk and burden by initiating their study first in a cohort of patients with prolonged injury. With these dimensions mapped they might then have moved to patients with more recent injury to explore dimensions that require an efficacy signal.

A third common challenge arises in studies involving high-risk interventions. Trials testing biologics for treating brain disorders are one example briefly visited in the previous section. In contrast to dose escalation trials, these studies typically explore very few intervention ensembles. This parsimonious approach to exploring dimension variables may actually lead to early study inefficiencies. For instance, in a Parkinson’s disease gene transfer candidate phase 1 trial, investigators varied one dimension value, dose, and only by a single increment [25]. The subsequent randomized trial failed to meet its primary endpoint, though it trended positively and met secondary endpoints [25]. Should the study count as a negative one, and the candidate be redesigned, or does the hint of signal support new trials? The authors of the report suggested the phase 2 study might have failed to implement the intervention in the optimal way: the 12-month follow-up period for their primary endpoint may have provided insufficient time for disease response to emerge. They further speculated that delivery to an additional brain target, the substantia nigra, might have improved response. Clearly, then, the boundaries on the values for two dimensions (earliest detection of efficacy and delivery target) remain unanchored. A larger, more systematic exploration of these dimensions in a phase 1 trial might have provided a better basis for identifying an intervention ensemble that produced efficacy signal in phase 2, hence ultimately sparing more patients of the burdens of an ineffective intervention ensemble.

I close by discussing a few further implications, and limitations of the framework offered above. Early studies should consider ways to maximize the efficiency of exploring dimensions within a broader program of research. Successful translational research programs will eventually discover necessary operation dimensions, but they may require more patients than necessary if this discovery process is spread out over numerous trials that are not well coordinated with each other. One option is to consider designing phase 1 studies that systematically explore a larger number of dimensions. Another option is to design studies that explore a diverse space of combined dimensions. For example, Piantadosi describes two-drug combination dose-finding trials that map a hypothetical ‘response surface’ by administering factorials of two drugs at several doses. This approach, if adaptively applied, can have advantages in terms identifying optimal combination doses with maximum efficiency [10]. Such designs might be extended for other dimensions within an intervention ensemble; I leave to others how such multifactorial experiments might be designed and reconciled with statistical efficiency.

The framework of early human studies also offers predictions. First, late human trials that apply intervention dimension values that reside beyond the boundaries of previously validated intervention ensembles are more likely to fail translation than trials that employ interventions within boundaries. This may seem trivial. However, it is an open question how frequently later trials use intervention ensembles where there is evidence to suggest they reside beyond the boundaries of a promising intervention ensemble. Second, completion of translation trajectories is likely to be more efficient where they begin by carefully mapping dimensional boundaries. All else being equal, trajectories that have not mapped boundaries early on should require more phase 3 trials to produce demonstrable evidence of therapeutic value. Third, healthcare systems are likely to encounter greater inefficiency in applying interventions where dimensional boundaries have not been well defined in early human studies as compared with interventions where they have been well defined. These inefficiencies would be reflected in relabeling of drugs, or widespread applications of an intervention that are off label and not well supported by clinical evidence. With some additional work, each of these predictions can be formulated as a testable hypothesis.

An important limitation of the above framework is its ideality. Drug development is constrained by many non-epistemic variables, including financial and recruitment difficulties. The framework offered here provides little guidance about how evidentiary aims ought to be pursued within the practical constraints of drug development. If a biotechnology company can only budget for a single phase 1 trial, perhaps it makes ethical sense to ‘bet the house’ on one or two intervention ensembles rather than systematically explore others. I leave for a later analysis how this framework is reconciled with these serious practical challenges. A second limitation is that boundaries are often resolved at later stages of drug development, as when biomarkers of response are identified in large populations. Therefore, the framework provided above is, to a lesser degree, also applicable in later stages of drug testing.

And there are unsettling implications as well. This framework undermines the position, frequently mooted in the trial ethics literature, that early human trials pursued in desperately ill volunteers can be viewed as therapeutic endeavors. However, burdens entailed in some early human trials are substantial. For instance, the probability of drug-related death in cancer phase 1 trials is 0.5% [26], and almost the same for fatal cerebral hemorrhage in Parkinson’s cell transplantation studies [27]. If one accepts the views that: (a) advancing drug translation with reasonable efficiency is simply impossible without designing trials that define boundaries, and (b) therapeutic translation should be prosecuted with reasonable efficiency, then the framework suggests that parties to drug translation must come to terms with the position that studies that expose patients to nontherapeutic research procedures that carry substantial risk of death can, at least in special circumstances, be ethical.

These concerns and limitations aside, this analysis extends our understanding about the aims and architecture of early human trials within the context of a broader program of research. A clear description of the framework supporting early trials is critical for meaningful progress in resolving persistent ethical and methodological debates surrounding such studies.

## Competing interests

The author declares he has no competing interests.
